# Salinomycin exerts anti-angiogenic and anti-tumorigenic activities by inhibiting vascular endothelial growth factor receptor 2-mediated angiogenesis

**DOI:** 10.18632/oncotarget.8555

**Published:** 2016-04-02

**Authors:** Tao Li, Xiaoxia Liu, Qin Shen, Wenjun Yang, Zhenghao Huo, Qilun Liu, Haiyan Jiao, Jing Chen

**Affiliations:** ^1^ Department of Oncology, General Hospital of the Ningxia Medical University, Yinchuan 750004, China; ^2^ Department of Medical Genetic and Cell Biology, Ningxia Medical University, Yinchuan 750004, China; ^3^ Key Laboratory of Fertility Preservation and Maintenance (Ningxia Medical University), Ministry of Education, Yinchuan 750004, China

**Keywords:** gastric cancer, salinomycin, angiogenesis, tumor growth, vascular endothelial growth factor 2

## Abstract

Anti-angiogenesis targeting VEGFR2 has been an attractive strategy for cancer therapy for its role in promoting cancer growth and metastasis. However, the currently available drugs have unexpected side effects. Therefore, development of novel VEGFR2 inhibitors with less toxicity would be of great value. In this study, we describe a novel and safely VEGFR2 inhibitor, Salinomycin (Sal), which was screened from the drug libraries of Food and Drug Administration (FDA) and prohibited the binding of the ATP at its binding pocket of VEGFR2 using molecular docking model. Sal could interfere a series of VEGF-induced angiogenesis processes including proliferation, migration, and tube formation in HUVECS *in vitro*. Matrigel plug model demonstrated Sal strongly inhibited angiogenesis *in vivo*. We found that Sal significantly decreased VEGF-induced phosphorylation of VEGFR2 and its downstream STAT3 in dose- and time-dependent manner in HUVECs. Besides, Sal could directly reduce the cell viability and induce apoptosis in SGC-7901 cancer cells *in vitro*. Sal inhibited constitutive STAT3 activation by blocking its DNA binding and reduced various gene products including Bcl-2, Bcl-xL and VEGF both at mRNA and protein levels. Intra-peritoneal injection of Sal at doses of 3 and 5 mg/kg/day markedly suppressed human gastric cancer xenografts angiogenesis and growth without causing obvious toxicities. Taken together, Sal inhibits tumor angiogenesis and growth of gastric cancer; our results reveal unique characteristics of Sal as a promising anticancer drug candidate.

## INTRODUCTION

Gastric cancer is one of the most common digestive malignant neoplasms worldwide [1, 2]. Despite considerable improvements that have been achieved through systemic therapy, the mortality rate of gastric cancer remains extremely high, and relapse and metastases occur in most cases [3]. Therefore, safer and more effective approaches are needed in gastric cancer therapy.

It is now well accepted that angiogenesis is a rate-limiting step in tumor progression, and provides a route for tumor metastasis [4, 5]. Deciphering the molecular mechanisms of tumor angiogenesis has recently allowed successful translation into clinical applications. When vascular endothelial growth factor (VEGF), a pro-angiogenic cytokine, specifically binds to distinct receptor tyrosine kinases (RTKs) like VEGFR1 (Flt-1), VEGFR2 (KDR/ Flk-1), and VEGFR3, it can exhibit its biological functions [4, 6]. Out of these kinases, VEGFR2 plays a major role in transducing angiogenic signals [7]. The autophosphorylation of Tyr1175 on VEGFR2 results in the activation of downstream signaling events. Amongst the endothelial cell signaling cascades, signal transducer and activator of transcription 3 (STAT3) is frequently associated with the transformation and progression of various human malignancies. The activated STAT3 forms homodimers and is then translocated into the nucleus to regulate the expression of target genes involved in cell proliferation (e.g., cyclinD1), survival (e.g., BCL-2, BCL-xl), invasion (e.g., matrix metalloproteinase-9), and angiogenesis (VEGF) [8]. Aberrant activation in the VEGFR2 signaling pathway contributes to cell differentiation, proliferation, metastasis, apoptosis, angiogenesis, and inflammation [9]. At present, several targeting VEGFR2 compounds have been used in clinic, including FDA approved drugs such as sorafenib, sunitinib, and vandetanib. Moreover, numerous small molecule VEGFR2 inhibitors are under clinical and preclinical evaluation, such as YLT192 [10] and SKLB261 [11]. However, adverse effects have been observed, indicating that development of much more safer VEGFR2 inhibitors is still needed.

**Figure 1:**
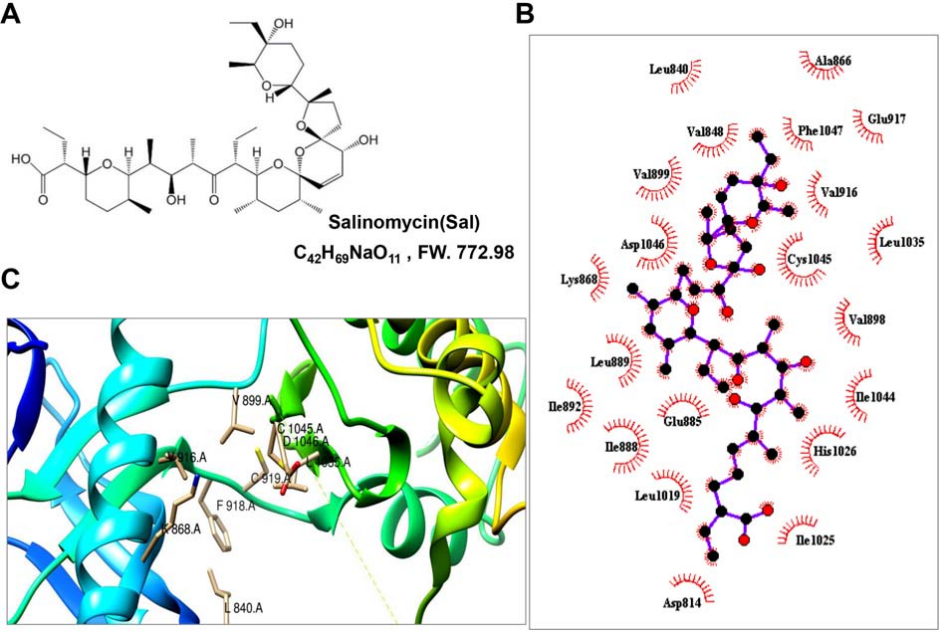
Sal interacted with the ATP-binding sites of VEGFR2 kinase domain (**A**) Chemical structure of Sal. (**B**) 2-dimensional interaction map of Sal and involved amino acids of VEGR2 proteins were calculated by LigPlot Software. Key describes the types of involved interaction and bonds. (**C**) ribbon structure of VEGFR2 protein in green color has been created by Chimera program. ATP binding site of VEGFR2 crystal has been shown.

Salinomycin (Sal) (Figure 1A), a carboxylic polyether ionophore isolated from *Streptomyces albus*, has been used extensively as an agricultural antibiotic to prevent coccidiosis in poultry [12]. Recent studies have shown that Sal displays potent anti-tumor activities in different types of human cancer stem cells (CSCs) [13, 14], including colorectal-[15], lung-[16], gastric-[17], pancreatic-[18], and osteosarcoma CSCs [19]. It also can kill cancer cells, including those of colorectal-, prostate-, breast-, ovarian-, hepatocellular- and chemotherapy-resistant cancer cells and so on [20–29]. Sal kills these cells most likely by increasing DNA damage [30, 31], up-regulation of death receptor-5 [27], restoring normal drug sensitivity in cancer cells [32], affecting the epithelial-mesenchymal transition, and activating autophagy, mitophagy and mitochondrial polarity [33–35] through activating AMP-activated protein kinase [36], or inhibiting β-catenin/TCF complex association *via* FOXO3a activation [37], Wnt/β-catenin [38–40], STAT3/Skp2 [41], Akt/NF-κB/mTOR [42, 43] signaling pathways. However, the role of Sal in tumor angiogenesis and the related molecular action have not been clearly elucidated. In this article, we evaluated the anti-angiogenic and anti-tumorigenic activities of Sal in gastric cancer and the involved molecular mechanism *in vitro and vivo*.

## RESULTS

### Salinomycin was located at the ATP-binding sites of the VEGFR2 kinase domain

Using molecular docking analysis, a drug named salinomycin (Figure 1A), which targeted the VEGFR2 protein, was screened from the drug libraries of FDA. We assessed the binding pattern between the x-ray crystal structure of VEGFR2 and Sal. Through analysis of the Sal docking results, the results showed that Sal could occupy the activity pocket. The amino residues Leu 840, Val848, Val899, Asp1046, Lys868, Leu889, Ile892, Ile888, Leu1019, Glu885, Asp814, Asp814, His1026, Leu1035, Phe1047, Glu917 and Ala866 interacted with the protein through the hydrophobic interaction (Figure 1B). And the amino residues Glu885, Ile1025, Ile1044, Val898, Cys1045 and Val916 interacted with the protein through hydrophilic interaction (Figure 1B). Among them, the residues of K868, V916, L840, L1035, V899, C1045 and D1046 could interact with both of adenosine triphosphate (ATP) and Sal compound by different interactions (Figure 1C). Such binding pattern of Sal with VEGFR2 may prohibit the binding of the ATP at its binding pocket.

### Salinomycin inhibited endothelial cell viability

**Figure 2:**
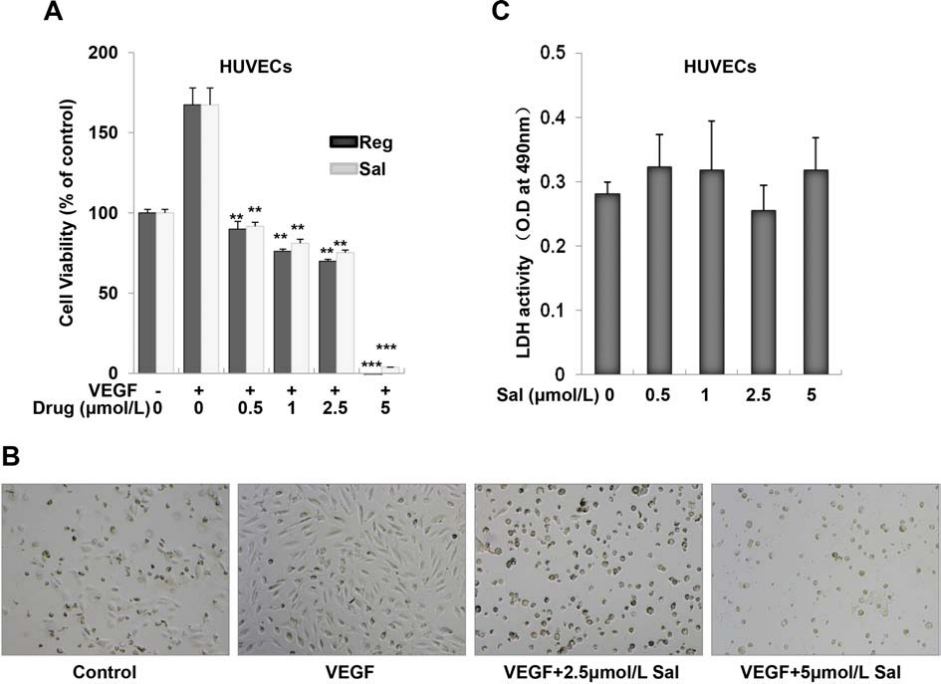
Sal inhibits VEGF-induced cell growth in HUVECs (**A**) Both Sal and Regorafenib inhibited VEGF-induced proliferation of endothelial cells dose-dependently. Cell viability was determined by MTS assay as described in the Materials and Methods. (**B**) Representative images were photographed (magnification at 40×). (**C**) Sal treatment did not result in LDH release from endothelial cells using LDH cytotoxicity assay kit, indicating that Sal exerts little cytotoxicity effect on HUVECs. *Columns*, mean from three independent experiments with triplicate. **P < 0.01; ***P < 0.001 versus VEGF control.

Cell viability was examined using MTS assay. As showed in Figure 2A and 2B, the proliferation of endothelial cells induced by VEGF was decreased in a dose-dependent manner after Sal or Regorafenib treatment in the range of 0.5–5 μM for 72 h, indicating the inhibitory effects of these two inhibitors dependent on VEGF-induced HUVECs proliferation. Especially, both of them showed similar activity, with the half maximal inhibition concentration (IC_50_) of 2.5 μM. To further examine whether Sal would result in toxic effects in HUVECs, lactate dehydrogenase (LDH) cytotoxic assays were carried out. As shown in Figure 2C, at the effective concentration of 0.5–5 μM, Sal caused minimal toxicity in HUVECs.

### Salinomycin inhibited VEGF-induced endothelial cell migration and tube formation in HUVECs

**Figure 3:**
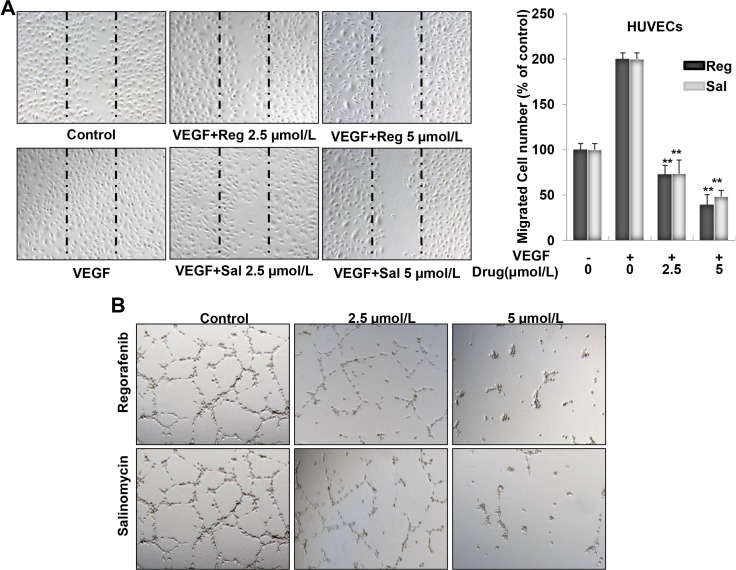
Sal inhibits VEGF-induced migration and tube formation in HUVECs (**A**) Both Sal and Regorafenib remarkably inhibited VEGF-induced endothelial cells migration in wound healing assay. Cells were wounded with pipette and treated with vehicle or indicated concentrations of Sal or Regorafenib. After 7–9 h, the migrated cells were quantified by manual counting. (**B**) Both Sal and Regorafenib inhibited the tube formation of endothelial cells. After treated with vehicle or indicated concentrations of Sal or Regorafenib for 8–10 h, representative fields in each group were presented (magnification at 100×). *Columns*, mean from three independent experiments with triplicate. **P < 0.01; ***P < 0.001 versus VEGF control.

Cell migration is an essential step in angiogenesis. Thus, we investigated the effects of Sal *vis-a-vis* Regorafenib on the chemotactic motility of endothelial cells using a wound-healing assay. The results showed that Sal and Regorafenib concentrations ranging from 0.5–5 μM, significantly inhibited the migration of VEGF-induced HUVECs in a dose-dependent manner (Figure 3A). The inhibitory efficacy of Sal was similar with that of Regorafenib. Then, we tested the effect of Sal and Regorafenib on capillary-like tube formation in HUVECs. When HUVECs were seeded on Matrigel, robust tubular-like structures were formed in the vehicle group within 8–10 h (Figure 3B). As shown in Figure 3B, almost 80% of the tube network was destroyed when HUVECs were incubated with either Sal or Regorafenib at 5 μM.

### Salinomycin inhibited neovascularization *in vivo*

**Figure 4:**
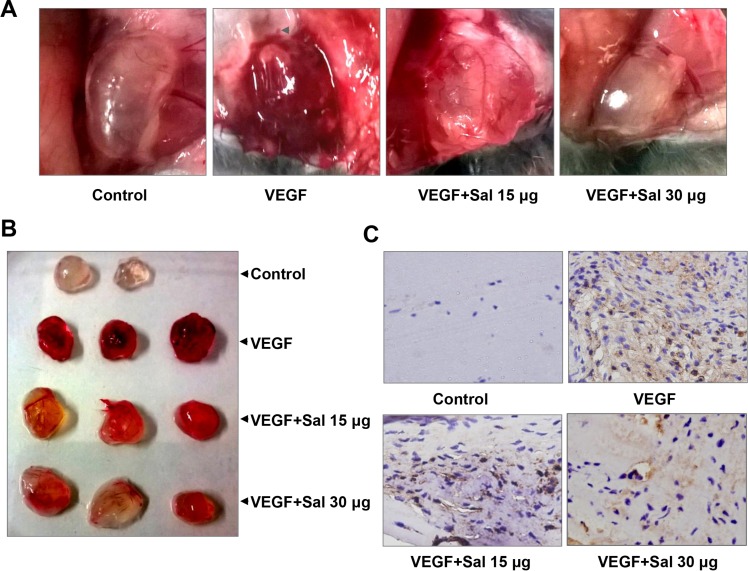
Sal inhibits VEGF-induced angiogenesis *in vivo* (**A** and **B**) representative images of Matrigel plugs in each group (*n* = 4~6). (**C**) immunohistochemistry analysis with CD31 antibody was performed on the sections of Matrigel plugs (magnification, 400×), showing CD31-positive endothelial cells.

We further measured the *in vivo* anti-angiogenic activity of Sal by a Matrigel plug assay. As shown in Figure 4A, Matrigel plugs containing VEGF alone appeared dark red, indicating that functional vasculatures had formed inside the Matrigel *via* angiogenesis triggered by VEGF. In contrast, the addition of different amounts of Sal (15 or 30 mg per plug) to the Matrigel plugs containing VEGF dramatically inhibited vascularization, as shown in Figure 4A. These plugs displayed a much paler appearance (Figure 4B). Immunohistochemical staining indicated that a large number of CD31-positive endothelial cells existed inside the plugs with VEGF alone, whereas the number of CD31-positive endothelial cells in Sal-treated groups decreased dramatically (Figure 4C). These results indicated that Sal inhibited VEGF-induced angiogenesis *in vivo*.

### Salinomycin attenuated VEGFR2 tyrosine kinase activity and VEGFR2-mediated STAT3 signaling pathways in endothelial cells

**Figure 5:**
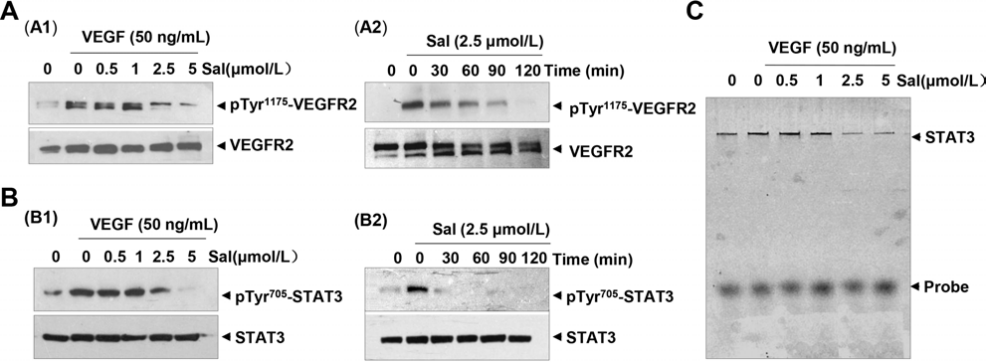
Sal inhibits VEGFR-mediated STAT3 cascade in endothelial cells (**A** and **B**) Sal dose- and time-dependently suppressed the activation of both VEGFR2 (Tyr^1175^) and downstream STAT3 triggered by VEGF in endothelial cell by Western blotting analysis. (**C**) Sal dose-dependently inhibited VEGF-induced DNA binding activity of STAT3 in endothelial cells. Nuclear extract was prepared and examined by EMSA assay. Three independent experiments were performed.

It is known that VEGF signaling events relevant to tumor angiogenesis are mainly mediated by VEGFR2 phosphorylation. The binding of VEGF to VEGFR2 leads to the activation of various downstream signaling molecules responsible for endothelial cell proliferation, migration, tube formation, and survival. In present studies, we found that Sal, at concentrations ranging from 0.5 to 5 μM, inhibited the phosphorylation of VEGFR2 and downstream STAT3 in HUVECs in a dose- (Figure 5B1) and time- (Figure 5B2) dependent manner. In contrast, total levels of VEGFR2 and STAT3 were not affected by Sal treatment. Additionally, we performed additional experiments and investigated whether Sal affected VEGFR1 activity. We found that Sal had little effect on the constitutive phosphorylation of VEGFR1 under the same conditions (Supplementary Figure 3). After being activated by VEGF, activated STAT3 forms homodimers or heterodimers, then translocates into the nucleus to result specific DNA binding to the promoters of target genes and thereby induced unique gene expression programs. The result of an electrophoretic mobility shift assay (EMSA) confirmed that treatment with Sal dramatically blocked this process and led to the dose-dependent inhibition of STAT3 DNA binding activity in HUVECs (Figure 5C). These data indicated that in addition to the blockade of constitutive STAT3 activation, Sal also exerted inhibitory effects on irreducible STAT3 activity.

### Salinomycin inhibited STAT3 signaling in SGC-7901 cells

Our study demonstrated that Sal exerts antiangiogenic activity *in vitro* and *in vivo* through blocking VEGFR2/STAT3 pathway in endothelial cells, suggesting that STAT3 is a potential target of Sal in gastric cancer cells. To address such a possibility, we examined the inhibitory effect of Sal on STAT3 in human gastric cancer SGC-7901 cells. The results showed that Sal decreased the phosphorylation of the STAT3 protein (at Y705) (Supplementary Figure 1A), blocked DNA binding ability of STAT3 (Supplementary Figure 1B) and modulated the expression of the anti-apoptotic genes (Bcl-2 and Bcl-xL) and the angiogenic gene product (VEGF) (Supplementary Figure 1C). The mechanism was reportedly regulated by STAT3, at much higher effective concentration ranging from 10 to 15 μmol/L. Similarly, we also observed that secreted VEGF by SGC-7901 was also dose-dependently inhibited by Sal analyzed by Elisa assay (Supplementary Figure 1D).

### Salinomycin inhibited tumor growth *in vitro*

Since Sal suppressed the activation of STAT3 and STAT3-regulated proliferative gene products, we further explored the antiproliferative activity of Sal, a broad of gastric cancer cell lines were treated with serial concentrations of Sal by the MTS assay. Following 72 h exposure to Sal, a dose-dependent growth suppression was observed in all cancer cells, with an IC_50_ value ranging between 10 and 15 μmol/L (Supplementary Figure 2A). Similar findings also were confirmed by Annexin V/propidium iodide staining assay (Supplementary Figure 2B) in accompany with an increased expression of cleaved caspase-3 in treatment of SGC-7901 cells 72 hours later (Supplementary Figure 2C). In addition, we further investigated the cytotoxicity of Sal on normal gastric epithelial cells (GES-1). Our results showed that Sal inhibited the proliferation of GES-1 cell with an IC_50_ over 20 μmol/L (Supplementary Figure 2A), which is much higher than that observed in gastric cancer cells. Together, these data suggested that Sal could induce apoptosis in gastric tumor cells with low side effect.

### Salinomycin inhibited tumor growth and angiogenesis in a human gastric cancer xenograft mouse model

**Figure 6:**
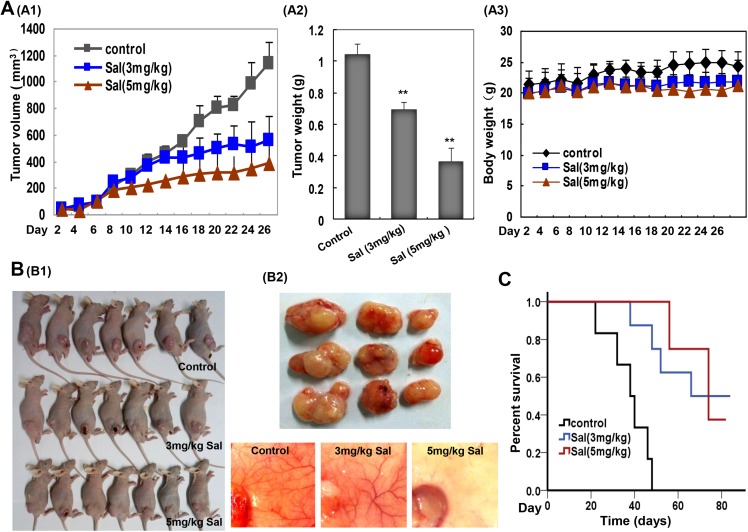
Sal suppresses tumor growth and angiogenesis in a human gastric cancer xenograft mouse model (**A**) Sal inhibited tumor growth as measured by tumor volume (**A1**) and tumor weight (**A2**) without detectable toxicity (**A3**) at the tested dose. *Columns and dots*, mean; *bars*, standard deviation; **P < 0.01; ***P < 0.001 versus the control group. (**B**) Sal inhibits both solid tumors (**B1**) and neovascularization (**B2**). (**C**) Kaplan-Meier survival curve for Sal treated mice in comparison to control group. Sal prolonged the life span of mice.

Further, we evaluated the *in vivo* anti-angiogenic and anti-tumorigenic activities of Sal using a xenograft model. Once a tumor size of 120 mm^3^ was achieved, mice were injected with vehicle (control), or vehicle with Sal. The used dosage of Sal was chosen according to published literature [44, 45]. As shown, intraperitoneal administration of Sal (3 and 5 mg/kg/d, 28 days) significantly reduced tumor volume (Figure 6A1) and tumor weight (Figure 6A2). The percent of tumor growth inhibition (TGI) of Sal was 51.3% and 66.3% at 3 and 5 mg/kg/d groups. Furthermore, Sal treatment was well tolerated, and there was no significant difference in weight loss in all groups during Sal treatment periods (Figure 6A3). Moreover, when the skin of each mouse was pulled back to expose an intact tumor, we found that Sal-mediated suppression of tumor growth was well correlated with angiogenesis inhibition, as shown in the representative image from each group (Figure 6B1 and 6B2). Additionally, we assessed whether Sal treatment would prolong the life span of mice. As a surrogate of survival, mice were sacrificed when tumor reach approximately 1,500 mm^3^ in any one dimension. A Kaplan-Meier plot for the time course of survival showed that Sal-treated mice survived for up to 80 days compared to the normal group (Figure 6C).

**Figure 7:**
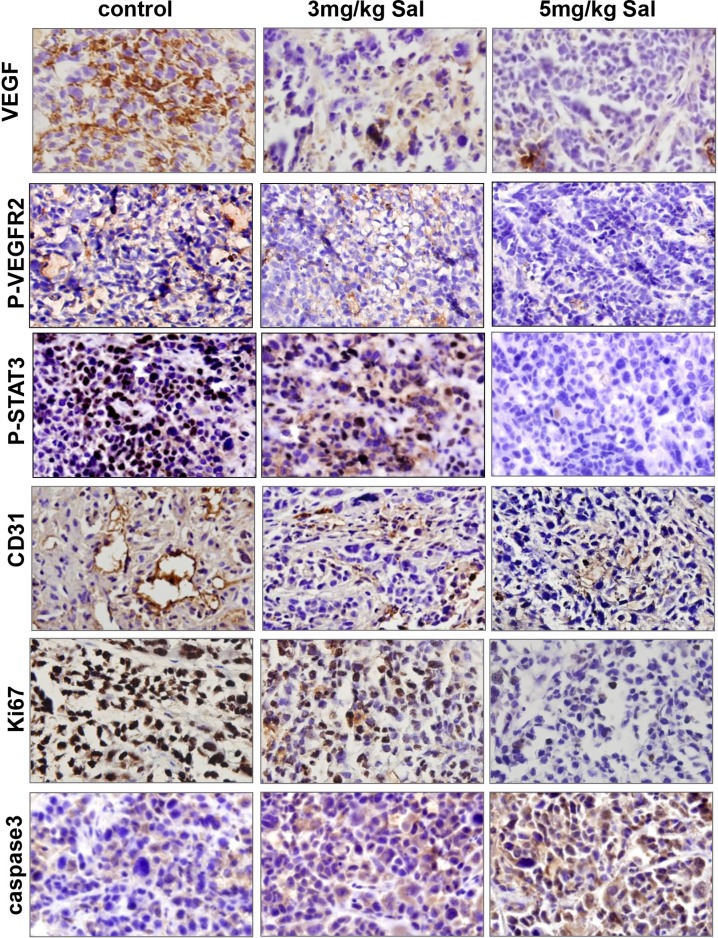
Mechanism analyses of xenograft tumors VEGF, p-VEGFR2, p-STAT3, CD31, Ki67 and caspase3 immunohistochemical staining analysis revealed that Sal inhibited angiogenesis, tumor proliferation and induced apoptosis in human gastric cancer xenografts through VEGFR2/STAT3 signaling pathway.

To better understand the mechanism of antitumor activities *in vivo*, we further carried out immunohistochemical analysis using tumors tissues at the end of the treatment. As shown in Figure 7, Sal dramatically regulated the protein expressions of markers for both cell proliferation (Ki67 staining) and apoptosis (caspase-3 staining) in the treatment groups as compared with the controls. To further investigate whether Sal inhibited tumor growth by suppressing tumor angiogenesis, immunostaining for specific proteins was performed. Our results showed that the number of CD31-positive endothelial cells and the expression levels of VEGF, p-VEGFR2, and p-STAT3 were all significantly decreased. Collectively, these results indicated that Sal-mediated suppression of SGC-7901 xenograft growth *in vivo* was associated with decreased neovascularization and proliferation as well as increased apoptosis index.

## DISCUSSION

Angiogenesis inhibition has become an important strategy for cancer therapy. More and more angiogenesis inhibitors have been used in the clinic. These include monoclonal antibody agents or small-molecule drugs, which target VEGF ligands or VEGFR. However, their success is insufficient and several issues have arisen from their applications. They elicit some side effects, even increase metastasis, and possibly develope treatment resistance [46]. Hence, there is an urgent need to find new anti-angiogenic inhibitors that can be more efficacious and less toxic for cancer therapy, particularly agents that exhibit activity against drug resistance and/or metastasis.

In this study, we report a significant finding that Sal, a widely used agricultural antibiotic drug approved by FDA, can directly act on both tumor endothelial cells and tumor cells. We clearly demonstrated Sal inhibited various aspects of angiogenesis including endothelial cell proliferation, migration and capillary structure formation *in vitro* at relatively lower concentration. Sal significantly inhibited neovascularization by matrigel plug assay *in vivo* in a dose-dependent manner. Previous studies have suggested that phosphorylation of VEGFR2 is critical for VEGF-mediated neovascularization [5]. Here, we found that Sal affects the multiple facets of vascular endothelial angiogenic signaling through VEGFR2, *via* prohibiting the binding of the ATP at its binding pocket of VEGFR2 using molecular docking assay and decreasing VEGF-induced phosphorylation of VEGFR2 ^(Tyr1,175)^ expression without affecting the activation of VEGFR1 as observed by western blotting and immunohistochemistry. The results suggested the possibility that Sal exerts its anti-angiogenic effect preferentially *via* VEGFR2 signaling pathway. Many kinase inhibitors could exert their inhibitory effects through purely or partially competing against ATP and subsequently suppressing the receptor autophosphorylation [47, 48]. They were acting as ATP minetics that bound to this site and competed with cellular ATP. Using computational modeling, we found that Sal may directly bind to VEGFR2 domain and could stably locate at the ATP-binding pocket. There are seven amino acids at the ATP pocket, which were essential for the stable conformation of VEGFR2/Sal complex. Rest amino acids can interact with the protein through the hydrophobic or hydrophilic interactions. All the unique binding modes largely promoted the conformational stability of the Sal/VEGFR2 complex, which interfered the binding of VEGF to VEGFR2. Additionally, we found that Sal also could indirectly reduce the paracrine secretion of VEGF from tumor cells, which further strengthens its anti-angiogenic activity.

Apart from the here described newly discovered anti-angiogenic effect, our observation also showed Sal had anti-tumorigenic effects. In current study, we confirmed that Sal caused the inhibition of proliferation, and induced substantial apoptosis in tumor cells by prohibiting constitutive STAT3 activation in SGC-7901 cells and thereby its DNA binding ability. Sal further reduced the expressions of STAT3-modulated Bcl-2 and Bcl-xL both at mRNA and protein levels, and increased the levels of pro-caspase-3 all known to promote tumor survival and tumor growth. Previous studies showed that Sal is one ionophore with specificity for K^+^ ions and can promote hyperpolarization of mitochondria [12]. These effects on mitochondrial polarity leading to altered metabolic dependence of cancer cells could be one reason for additive cell death effect of Sal. On the other hand, several reports demonstrated that autophagy is clearly linked to cell death [49]. Sal can induce autophagy in breast and colon cancer cell lines [33, 34], concomitant to the induction of reactive oxygen species, which may be another potential mechanism of caspase-independent cell death caused by Sal.

Recently, Sal gained substantial attention when it was first identified as a drug preferentially killing cancer stem cells. Sal has been shown to overcome apoptosis-resistance in several types of cancer cells with far less side effects, and inhibits tumor metastasis and recurrence by disruption TGF-β1-induced epithelial-to-mesenchymal transition (EMT) and/or other signaling pathways [50, 51], which might help to prevent and/or delay treatment resistance in anti-angiogenic therapy. All these characteristics could be reasonable to make Sal distinct from present angiogenesis inhibitors; especially those used in the clinic, and become a promising anticancer drug candidate. However, further studies are needed to improve its biological activity and physicochemical properties.

## MATERIALS AND METHODS

### Chemicals and reagents

Reagents of Salinomycin (Sal) and Regorafenib (Reg) were purchased from Sigma (St. Louis, MO, USA) and Selleck Chemicals (Shanghai, China), respectively. Both of them was dissolved in 100% DMSO to form a 20 mM solution and stored at −80°C in small aliquots until needed. Growth factor-reduced Matrigel was purchased from BD Biosciences (San Diego, CA). Antibodies against VEGFR2 (2479^#^), STAT3 (9139^#^), Bcl2 (^#^2872), BCL-xl (^#^2764), Caspase-3(^#^9664), and phosphor-specific anti-VEGFR2 (Tyr^1,175^) (^#^2478) and anti-STAT3 (Tyr^705^) (^#^9145) were purchased from Cell Signaling Technology (Danvers, MA, USA). Anti-CD31 (^#^ab28364), anti-Ki67 (^#^ab66155), anti-VEGFR1 (ab32152^#^) and phosphor-specific anti-VEGFR1 (Y1213) (ab195762^#^) were provided by Abcam (UK). Recombinant human VEGF (VEGF_165_) (^#^293-VE-010) and VEGF ELISA kits (^#^DVE00) were purchased from R&D Systems (MN, USA). All other reagents were acquired from Sigma-Aldrich (St Louis, MO, USA).

### Cell culture

Primary human umbilical vein endothelial cells (HUVECs) were gifted from Dr Mingyao Liu (The Institute of Biomedical Sciences and School of Life Sciences, East China Normal University, Shanghai, China) and cultured in endothelial cell culture medium (ECM) as described previously [52]. Both AGS, a human gastric cancer cell line and GES1, a normal gastric epithelia cell line were purchased from American Type Culture Collection. Other SGC-7901, MGC-803, and BGC-823 cancer cell lines were obtained from the China Center for Type Culture Collection. All these cells were cultured in RPMI-1640 medium supplemented with 10% fetal bovine serum. Cells were maintained in a humidified incubator at 37°C with 5% CO_2_ and the medium was replaced every 48 h.

### Molecular docking

The docking essay was carried out to assess the binding pattern between VEGFR2 receptor and Sal by using Autodock 4.2 [53]. The ligands were drawn by using Chemoffice and transported to 3D structure by using Openbabel without structure optimization [54]. The receptor and ligands were prepared for docking by using the MGLTools1.5.6 [53], with adding Gasteiger Charges and polar hydrogen. The protein structure was obtained from Protein Data Bank (http://www.rcsb.org), and the water and salt ions were removed for the next step. For the docking parameters, the size of grid box was 56Å * 40Å * 40Å and the center of box was (−27.76, −0.681, −8.054). The graphics of molecule-protein interaction was showed in UCSF Chimera1.9 [55] and LigPlot [56] with default parameter.

### Cell viability assay

HUVECs or gastric cancer cell lines were seeded at 4.5 × 10^3^ to 5.5 × 10^3^ cells per well in 96-well culture plates and treated with or without VEGF (10 ng/mL) or increasing serial dosages of Sal (0, 0.5, 1, 2.5, 5, 10, and 15 μM) for 72 h. Regorafenib served as a positive control. Cell viability was determined using the MTS assay as described previously [52]. After 2 h of incubation, the absorbance was measured at 490 nm with a microplate reader (Bio-Red, USA). Three independent experiments were performed.

### Lactate dehydrogenase (LDH) toxicity assay

The LDH release assay was performed using a cytotoxicity detection kit plus (LDH) (Roche Diagnostics) according to the manufacturer's instructions. In brief, HUVECs were seeded in a 96-well plate at a density of 5 × 10^3^ cells per well. After incubation with various concentrations of Sal for 72 h, cell supernatants were collected and analyzed. The absorbance of formed formazan was read at 490 nm with a microplate reader (Biorad, USA). The LDH content of each sample was calculated according to the following formula: Cytotoxicity (%) = (experimental value − low control)/ (high control − low control) × 100. The assay was independently repeated three times.

### Endothelial cell migration assay

HUVECs (5 × 10^4^ cells per well) were allowed to grow to full confluence in 6-well plates pre-coated with 0.1% gelatin and then starved with basic ECM overnight. Thereafter, cells were wounded by scratching with pipette tips and washed with PBS. ECM supplemented with 0.5% FBS and different concentrations of Sal was added into the 6-well culture dishes. Regorafenib served as a positive control. Images of cells were taken using an inverted microscope (TE2000, Nikon, Japan) at 100× magnification after 7–9 h of incubation. The migrated cells were observed from three randomly selected fields and quantified by manual counting. Cells receiving only medium served as a vehicle control. Inhibition percentage was expressed as a percentage of the vehicle control (100%). The assay was independently repeated three times.

### Endothelial cell capillary-like tube formation assay

The tube formation assay was conducted as described previously [52]. After Matrigel polymerisation at 37°C for 1 h, HUVECs (8 × 10^4^ cells per well) were suspended in ECM and pre-treated with different concentrations of Sal or Regorafenib for 30 min, and then seeded onto the Matrigel. After 8–10 h of incubation, the network-like structures of endothelial cells were examined under an inverted microscope (TE2000, Nikon, Japan) at 100× magnification. Branching points in three random fields per well were quantified by manual counting. Cells receiving only ECM served as a vehicle control. Inhibition percentage was expressed as a percentage of the vehicle control (100%). The assay was independently repeated three times.

### Matrigel plug assay

As described previously [52], 0.5 mL of Matrigel in the presence or absence of 100 ng of VEGF, 20 units of heparin, and the indicated amount of Sal (15 and 30 μg) was subcutaneously injected into the ventral area of C57BL/6 mice (*n* = 3). Seven days after the implantation, intact Matrigel plugs were carefully removed. Those plugs were then fixed and embedded in paraffin. Specific blood vessel staining with CD31 antibody was carried out on 5 μm sections according to the protocol. Microphotographs were taken using an OLYMPUS BX41 photomicroscope (magnification at 400×).

### Western blotting analysis

To determine the effects of Sal on VEGFR2-mediated signaling cascades, HUVECs were first starved overnight in basic ECM. After being washed with fresh medium, cells were treated with Sal (0, 0.5, 1, 2.5, 10 and 5 μM) for 2–4 h, followed by stimulation with 50 ng/mL of VEGF for 10–15 min. Normal cultures of SGC-7901 gastric cancer cells were directly treated with indicated dilutions of Sal for 12 h. Whole cell lysates were prepared in RIPA buffer supplemented with PMSF and proteinase inhibitor before use. Proteins were separated by 6–10% SDS PAGE and transferred to polyvinylidene fluoride (PVDF) membranes. Membranes were then incubated with primary antibodies as described before [52]. Immunoreactive bands were then visualized by an enhanced chemiluminescence (ECL) detection system.

### Enzyme-linked immunosorbent assay (ELISA)

The concentrations of VEGF in Sal-treated SGC-7901 culture medium were measured using a Human VEGF ELISA kit according to the manufacturer's instructions (R & D Systems, Minneapolis, MN USA).

### RNA isolation and reverse transcription PCR

Total RNA from SGC-7901 cancer cells treated with different concentration of Sal for 72 h was extracted with the TRIzol reagent and converted to cDNA using a reverse transcriptase PCR (RT-PCR) kit (Thermo). Glyceraldehyde-3-phosphate dehydrogenase (GAPDH) was used as a loading control.

### Electrophoretic mobility shift assay

The DNA binding activity of STAT3 was examined by electrophoretic mobility shift assay using IRDye700 an infrared dye-labelled oligonucleotide probe (LI-COR, Biosciences) and analyzed in both HUVECs and SGC-7901 cells based on conditions defined by procedures from LI-COR.

### Annexin V/propidium iodide staining assay

Sal-mediated cell apoptosis was assayed by Annexin V/fluorescein isothiocyanate and propidium iodide staining (ApopNexin Annexin V FITC apoptosis kit; Millipore) as described in the instructions. Microphotographs were taken by an OLYMPUS BX41 photomicroscope (magnification at 200×).

### *In vivo* anti-tumour activity

Briefly, SGC-7901 cells (3.5 × 10^6^ cell per mouse) were subcutaneously implanted into the flanks of 6-week-old BALB/cA nude mice. When tumors grew to about 120 mm^3^, the mice were randomly divided into three groups (*n* = 9). They were then intraperitoneally treated with or without Sal (3 mg/kg and 5 mg/kg) every other day. The tumor volume was measured using a vernier caliper and calculated according to the modified ellipsoid formula: Tumor volume (mm^3^) = (length) × (width) ^2^ × 0.52. After 80 days of treatment, the mice were sacrificed and whole tumor tissues were harvested, weighed, and photographed. Excised tumors were fixed in 10% formaldehyde and embedded in paraffin for immunohistochemical observation. Sections with a thickness of 5 μm were stained with antibodies against CD31, VEGF, p-VEGFR2, p-STAT3, and caspase-3. Microvessel density was calculated using ImageJ software (NIH, Bethesda, MD). Survival was evaluated by the Kaplan-Meier method. Microphotographs were taken using an OLYMPUS BX41 photomicroscope (magnification at 400×). All procedures used for animal experimentation were approved by the Institutional Animal Ethics Committee.

### Statistical analysis

Statistical comparisons between groups were conducted using one-way ANOVA followed by the Dunnet test. Data were presented as means ± SDs. *P* values of 0.05 or less were considered statistically significant.

## SUPPLEMENTARY MATERIALS FIGURES


