# Supplementary material for: Salinomycin exerts anti-angiogenic and anti-tumorigenic activities by inhibiting vascular endothelial growth factor receptor 2-mediated angiogenesis
Source: Oncotarget. 2016 Apr 2;7(18):26580–92. doi: 10.18632/oncotarget.8555 (PMC5042000; doi:10.18632/oncotarget.8555)
Supplement: Supplementary file 1 [file oncotarget-07-26580-s001.pdf]

Salinomycin exerts anti-angiogenic and anti-tumorigenic activities by inhibiting vascular endothelial growth factor receptor 2-mediated angiogenesis

Supplementary Materials

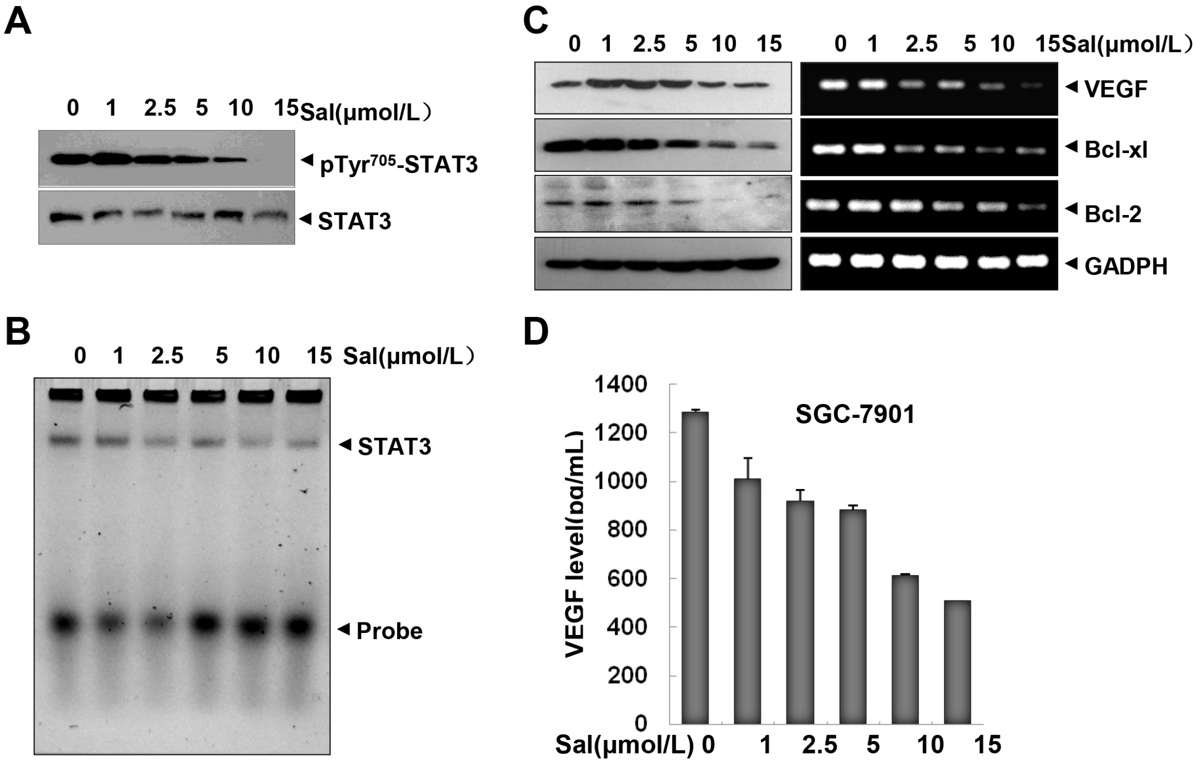

Supplementary Figure S1: Salinomycin blocks STAT3 signaling and suppresses STAT3 transcriptional activity in gastric cancer cells. (**A**), salinomycin concentration-dependently inhibited the activation of STAT3 in gastric tumor cells. (**B**), salinomycin inhibits STAT3 DNA binding in a dose-dependent manner in SGC-7901 cells analyzed by EMSA assay. (**C**), salinomycin suppressed the expression of mRNA (left) and protein (right) of Bcl-2, Bcl-xL and VEGF in SGC-7901 cells. (**D**), salinomycin dose-dependently inhibited secreted VEGF by SGC-7901 by Elisa assay. GAPDH were used as internal controls. Three independent experiments were performed.

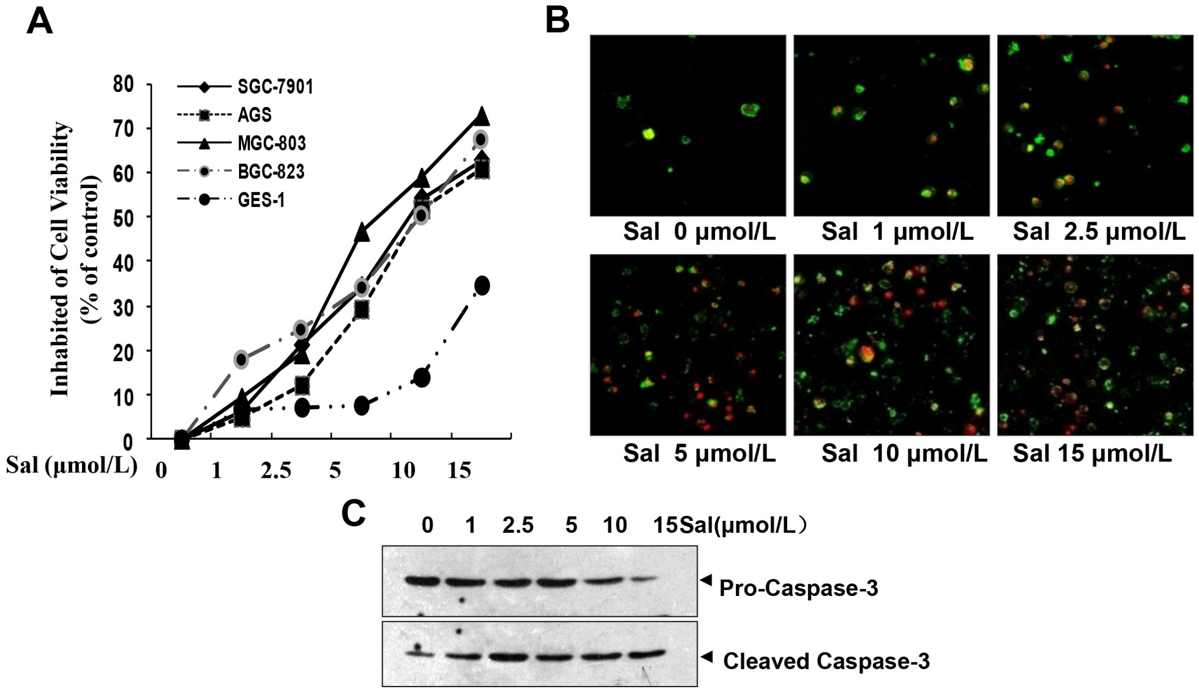

Supplemental Figure 2: Salinomycin inhibits proliferation and induces apoptosis of gastric cancer cell lines. (**A**), Salinomycin significantly suppressed viability of several gastric cancer cell lines and showed a low inhibitory effect on GES-1 measured by MTS assay. (**B**), treatment of salinomycin destroyed plasma membrane integrity of SGC-7901 cells confirmed by Annexin V/propidium iodide staining assay. (**C**), salinomycin dose-dependently induced cleavage of caspase-3 in cancer cells. Similar results were obtained in three independent experiments.

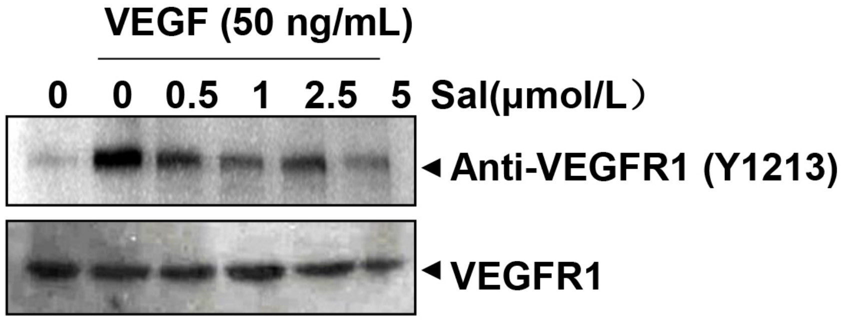

Supplementary Figure S3: Sal dose- dependently suppressed the activation of VEGFR1(Y1213). Cells were treated with Sal (0, 0.5, 1, 2.5 and 5μM) for 2–4 h, followed by stimulation with 50 ng/mL of VEGF for 10–15 min.
